# Assessing Trajectories and Bike Handling Abilities in Road Cycling with Global Positioning System Data

**DOI:** 10.3390/s25226977

**Published:** 2025-11-14

**Authors:** Andrea Zignoli

**Affiliations:** Department of Industrial Engineering, University of Trento, Via Sommarive 9, Povo, 38123 Trento, Italy; andrea.zignoli@unitn.it

**Keywords:** bike-rider model, optimal control, cycling computers, GPS trajectory fitting, roll angle estimation

## Abstract

In road cycling, developing bike handling skills can prevent crashes and falls. Nevertheless, bike handling remains largely unexplored in the world of road cycling. The goal of this research was to develop a methodology to assess bike handling during races and training by estimating the rider–bicycle roll angle and road-plane accelerations from global positioning system (GPS) data only. A multi-dimensional bike-rider mathematical model was included in an optimal control framework to follow a reference trajectory generated from GPS data points. Estimated variables and experimental data collected with a cost-effective setup showed good agreement, i.e., root mean square error (RMSE) of 12° and 0.1 g for roll angle and both longitudinal and lateral accelerations, respectively, in the worst-case scenarios. This methodology might allow for the estimation of key bike handling variables during fast segments with cost-effective instrumentation. It can therefore constitute a tool for objectively assessing bike handling in road cycling training and racing.

## 1. Introduction

Amid a rising number of crashes, the world of road cycling is calling for measures to improve riders’ safety conditions, and developing a rider’s technical skillset is an essential component of safe performances.

The forces that riders can generate while riding a bike are produced at the tyre–road interface (i.e., the contact patch). Contact forces can act both in the longitudinal and lateral directions, i.e., tangentially and orthogonally to the bike–rider system velocity. Longitudinal forces can be responsible for significant accelerations during propulsive and braking actions. Lateral forces cause the lateral accelerations while riders follow curved paths. When riders use the handlebars and their body weight to steer and lean into corners, the magnitude of the lateral forces is largely determined by the angular displacement of the bike–rider system about the longitudinal axis (i.e., the roll angle). The tyre–road interaction can only provide a maximum amount of grip force, and if the limit is exceeded suddenly or unexpectedly, the rider might lose control of the bike. A direct estimation of the magnitude of these forces can be obtained with the product of the vertical load acting on the tyre and the friction coefficient between the tyre and road surfaces. An indirect estimation of these forces can be provided by the longitudinal and lateral accelerations of the bike-rider system.

In the world of practice, bike handling refers to the performance of the rider-bike interaction, and much of the scientific research has been devoted to the design of guidelines to improve bicycle stability and manoeuvrability [1]. Bike handling remains, however, largely unexplored in road cycling races, especially from a sports performance perspective. Test batteries are the most common tools for objectively differentiating riders with good and bad bike handling [2]. It is very difficult for coaches to go beyond the results of the battery tests and provide the riders with indications about how to improve their bike handling and gain a competitive advantage.

Retrieving data that can be used to assess bike handling performance during road cycling races or training is highly problematic. In the world of both amateur and professional races, bikes are typically only instrumented with lightweight/compact cycling computers. These devices are equipped with global positioning system (GPS) receivers, and they are used to derive important information about cycling performance, such as total elevation gain and average speed. Cycling computers are usually not equipped with inertial measurement units (IMU), and they receive and fuse together information derived from both the GPS and an odometer positioned at the front wheel to achieve a reliable estimation of the travelling distance and speed in case of obstructions, e.g., tunnels, trees, buildings, clouds, etc. Cycling computers are often equipped with a barometer or/and a mono-axial inclinometer, which provides an estimation of the slope of the road. Given the absence of an onboard embedded IMU, information about the orientation of the bicycle is limited to the pitch (i.e., the angular displacement of the bike-rider system about its transversal axis) provided by the inclinometer (when present), and information about the roll angle and the lateral accelerations is usually not available.

Information about the longitudinal *a_x_* and lateral *a_y_* bicycle accelerations is particularly useful for bike handling assessment. For example, the g-g-diagram [3] consists of a graph where the lateral acceleration values are plotted against the longitudinal acceleration values. The limits of the friction forces are provided by the friction ellipse defined as follows:

(1) $${\left(\frac{a_{x}(s)}{\mu_{x}\;g}\right)}^{2}+{\left(\frac{a_{y}(s)}{\mu_{y}\;g}\right)}^{2}\leq 1,$$

where *μ_x_* and *μ_y_* are the tyre–road friction coefficients in the longitudinal and lateral directions, and *g* is the magnitude of acceleration due to gravity. Possible differences between *μ_x_* and *μ_y_* are due to the shape of the tyre–road contact patch, and they heavily depend on tyre shape and conformation (see, e.g., [4]). The shape of the distribution of the acceleration values inside the friction ellipse can be used to evaluate different riding styles, e.g., the combination of the acceleration values lying inside the adherence ellipse is considered safe. It is reasonable to think that the riders who can consciously and consistently ride close to the limits imposed by the friction ellipse without losing control of their bikes are the ones with better bike handling [5,6].

Several methods to estimate the longitudinal and lateral accelerations and the roll angle on two-wheeled vehicles with inexpensive hardware have been developed. For example, Boniolo et al. [7] and Lot et al. [8] developed a methodology to estimate the roll angle from gyroscopes and encoders on motorcycles. Gasbarro et al. [9] developed an algorithm for the reconstruction of the trajectory of a motorcycle starting from an onboard camera and accelerometers. Schlipsing et al. [10] developed a method to estimate the roll angle in motorcycles from greyscale images collected with an onboard camera. More recently, a cost-effective method to estimate the roll angle from a gyroscope on city bicycles has been proposed by Sanjurjo et al. [11]. In 2016, Cain [12] presented a new methodology where 11 wireless IMUs were used to sense the separated movements of the bicycle and rider. A Kalman filter was used by Cain to estimate the orientation of the bike-rider system. The method proposed by Sanjurjo et al. [11] requires a Kalman filter to be developed for the estimation of the bias in the roll angle measurements. Sanjurjo et al. [11] reported that their method (conceived for city bicycles) can provide a roll angle estimation with less than 2° of error. For the method proposed by Lot et al. [8] for motorcycles, the authors reported an error in the estimation of 4° [8]. Both Baniolo et al. [7], Lot et al. [8], and Sanjurjo et al. [11] adopted distance sensors to collect the gold-standard roll angle of the vehicle.

Although the methodologies differ in hardware configuration and data processing procedure, they all make use of a mathematical model of the bike-rider system dynamics. In these models, there is always a set of variables (i.e., the state variables) that can be used to describe the mathematical state of the system and to determine its future behaviour. To study bike handling, the roll angle and the lateral and longitudinal accelerations should be included in the set of state variables. If some of the state variables can be directly measured (e.g., position on the road plane from GPS), then experimental data can be incorporated into the model to estimate the unknown variables (e.g., roll angle, longitudinal and lateral accelerations) [13]. This process of estimating the unknown variables consists of finding the set of input variables (e.g., the steering angle or the power output) so the difference between the simulated and experimental data is minimised. The solution is often provided with dynamic optimisation techniques, such as optimal control [14,15,16].

The goal of this research was to develop a methodology for estimating roll angle and longitudinal and lateral accelerations in road cycling using GPS data only. A previously developed dynamic model of the bike–rider system [14] was implemented within an optimal control framework, where the control inputs are optimised to reproduce the measured GPS trajectories while remaining consistent with the system’s physical constraints. In this formulation, the optimal control solution inherently acts as a low-bandwidth filter, fitting noisy experimental data while enforcing dynamic feasibility. This approach allows reliable estimation of key motion variables even when the GPS signal is sparse or noisy, as often occurs in outdoor cycling environments. The model estimates were validated against experimental data collected in real-world conditions using a cost-effective setup consisting of a smartphone equipped with an IMU, demonstrating the potential for scalable application in training and competition scenarios.

## 2. Methods

A multi-dimensional model of bike-rider dynamics [14] was used in this study. An optimal control solver [17] was used to track the reference trajectory. The reference trajectory was computed by fitting the GPS data. Variables estimated with the model (roll angle and longitudinal and lateral accelerations) were compared to experimental data collected with a mobile phone equipped with an IMU. In this section, all the different elements of the analysis are discussed in detail.

### 2.1. The Rider–Bicycle Model

**Figure 1:**
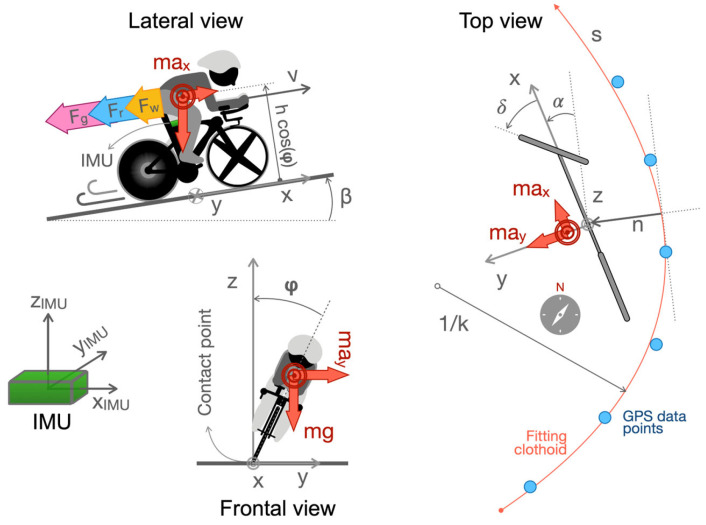
Schematic representation of the rider–bicycle model. The three axes x, y, and z are pointing forward, leftward, and upward, respectively. This also holds for the three axes of the inertial measurement unit (IMU): *x*_IMU_, *y*_IMU,_ and *z*_IMU_. **Lateral view**: resistance aerodynamic *F_w_*, rolling *F_r,_* and gravitational *F_g_* forces are reported. Longitudinal acceleration *a_x_* and the action of gravity are reported. The height of the centre of mass is *h,* and the slope of the road *β* is reported. The position of the IMU is highlighted. **Frontal view**: longitudinal acceleration *a_x_*, the action of gravity, and roll angle φ are reported. **Top view**: a generic clothoid (Euler spiral) fitting the global positioning system (GPS) points was used as the reference trajectory. The distance from the start of the clothoid is the curvilinear abscissa *s*, and the lateral distance from the clothoid *is n.* Vehicle heading *α*, steering angle *δ*, the action of the longitudinal *a*_x_ and lateral *a*_y_ accelerations, and the curvature radius 1/*k* of the reference trajectory are reported. The schematic compass indicates the true North.

The bike-rider system has been modelled as an inverted pendulum with a single mass point moving on a plane (i.e., *xy*) along a curve (Figure 1) and tilting with a roll angle *φ* [18]. The model has already been used along with an optimal control framework to solve pacing strategy optimisation problems [14,19], to design rider-assistance systems [20], to study cycling cornering strategies [21], and to evaluate cycling time trial performance [6]. Two inputs were used to control the model: the normalised steering angle *δ_n_* (between maximal and minimal values *δ_min_* and *δ_max_*) and normalised power output *W_n_* (between *W_min_* and *W_max_*).

The forces created at the tyre–road interfaces are applied to a single point of contact, i.e., the lower extremity of the pendulum at *z* = 0. The mass of the rider and the bicycle was set to 76 kg and 9 kg, respectively. In cycling, resistive forces have three main components [22]: (1) aerodynamic forces *F_w_* [23] were computed as half of the product of the air drag force coefficient *k*_v_ (0.15 kg/m, i.e., the product of a drag coefficient *C*_D_ of 0.7, a frontal area *A*_f_ of 0.35 m^2^ and an air density *ρ* of 1.23 kg/m^3^ [24]) and the squared longitudinal speed *v*; (2) the rolling resistive forces *F_r_* [25] were computed as the product of the rolling coefficient *C*_rr_ (0.004 [26]) and the mass of the system, the cosine of the road slope *β* and the constant of gravity *g*; (3) the gravitational force *F_g_* was computed as the product of the mass of the system *m*, the sine of the road slope *β* and the magnitude of acceleration due to gravity. Clothoids (i.e., Euler spirals) were used to fit the GPS data points and to define the reference trajectory of the rider–bicycle model. Clothoids are commonly used to describe curvilinear trajectories because their curvature *k* changes linearly with the curve length. This is extremely important because a linear change in the curvature allows for the definition of smooth transitions between paths, without abrupt changes in centripetal acceleration values. Curvilinear coordinates [27] were used to define the position of the bicycle: *s* constitutes therefore the longitudinal position, *n* is the lateral displacement of the centre of mass, *α* is the heading, and 1/*k* is the local radius of the single clothoid segment (top view, Figure 1).

### 2.2. The System of Equations

With the introduction of the curvilinear coordinates [27], the curvilinear abscissa was used as the independent variable instead of the time *t*. The equations of motion were obtained for the bike-rider system, and differential equations were solved in their explicit form to highlight the variations in the state variables with respect to *s*. The following substitution law was used:

(2) $$\frac{d}{ds}\dot{s}\left(s\right)=\frac{d}{ds}\frac{ds}{dt},$$

where the classic notation $\dot{x}$ indicates the time derivative of a generic variable *x*. This was adopted to improve the readability of the equations. 

The first equation of the dynamics defines the variation in α with *s*:

(3) $$\frac{d}{ds}\alpha \left(s\right)=\frac{\delta_{n}(s)\delta_{max}}{L\;\dot{s}(s)}-k\left(s\right),$$

where *L* is the wheelbase of the bike. The second equation of the dynamics defines the variation in *n* with *s*:

(4) $$\frac{d}{ds}n\left(s\right)=\frac{1}{\dot{s}(s)}(v\left(s\right)\sin\left(\alpha (s)\right))$$

The third equation of the dynamics defines the variation in v with *s*:

(5) $$\frac{m\;v\left(s\right)}{W_{max}}\frac{d}{ds}v\left(s\right)=\frac{W_{n}(s)}{\dot{s}(s)}-\frac{v\left(s\right)}{\dot{s}\left(s\right)W_{max}}\left(m\;g\;\left[C_{rr}\cos\left(\beta \left(s\right)\right)+\sin\left(\beta \left(s\right)\right)\right]+k_{v}{\left(v\left(s\right)-V_{w}(\alpha (s)\right)}^{2}\right),$$

where *V*_W_ is the wind velocity (which is dependent on the heading). The fourth equation of the dynamics defines the variation in *φ* with *s*:

(6) $$\frac{d}{ds}\varphi \left(s\right)=\frac{\dot{\varphi }(s)}{\dot{s}(s)}$$

The fifth equation defines the variation in the time derivative of *φ* with *s*:

(7) $$\frac{d}{ds}\dot{\varphi }\left(s\right)=\frac{h\;m\;g}{I_{X}g\;L\;\dot{s}(s)}\left({v\left(s\right)}^{2}\delta_{max}\delta_{n}\left(s\right)+L\;g\;\varphi (s)\right),$$

where *h* is the constant distance between the contact point on the road plane and the position of the centre of mass of the bike-rider system and *I_X_* is the moment of inertia of the system along the longitudinal direction. 

The sixth equation defines the variation of *W_n_* with *s*:

(8) $$\frac{d}{ds}W_{n}\left(s\right)=\frac{{\dot{W}}_{n}(s)}{\dot{s}(s)}$$

The seventh equation defines the variation of *δ_n_* with *s*:

(9) $$\frac{d}{ds}\delta_{n}\left(s\right)=\frac{{\dot{\delta }}_{n}(s)}{\dot{s}(s)}$$

The eighth equation defines the variation in the time derivative of *s* with *s*:

(10) $$\frac{d}{ds}\dot{s}\left(s\right)=\frac{v\left(s\right)\;cos(\alpha (s))}{1-n\left(s\right)k(s)}$$

The ninth equation defines the variation in t with s:

(11) $$\frac{d}{ds}t\left(s\right)=-\frac{1+k(s)n(s)}{cos(\alpha \left(s\right))v(s)}$$

Therefore, the vector of the state variables included {$\dot{s}\left(s\right),\;t\left(s\right),\;\delta_{n}\left(s\right),\;\dot{\varphi }\left(s\right),\;\varphi \left(s\right),\;v\left(s\right),\;n\left(s\right),\;\alpha \left(s\right)$}. The vector of the control variables included {$\delta_{n}\left(s\right),\;W_{n}(s)$}.

### 2.3. Optimal Control Problem Formulation

The main building blocks of an optimal control problem [17,28] are the system of equations (Equations (2)–(11) presented in the previous subsection), the objective function, the initial conditions, and the constraints.

The optimal control was used to find the combination of inputs that could minimise the objective function *J*. The objective function was defined as the weighted sum of three different goals:

(12) $$J=J_{1}+J_{2}+J_{3}$$

The first two terms (*J*_1_ and *J*_2_) include the discrepancies between experimental and simulated data (lateral displacement *n* from the fitting clothoid and longitudinal speed *v*):

(13) $$J_{1}=\int_{L_{0}}^{L_{f}}\frac{{\left(v(s)-v^{*}(s)\right)}^{2}}{{∆w_{v}}^{2}}\frac{1}{\dot{s}(s)}ds$$

(14) $$J_{2}=\int_{L_{0}}^{L_{f}}\frac{{\left(n(s)\right)}^{2}}{{∆w_{n}}^{2}}\frac{1}{\dot{s}(s)}ds$$

The third term (J_3_) includes the rate of change in the steering angle $\dot{\delta }$ and the rate of change in the power output $\dot{W}$ [14]:

(15) $$J_{3}=\int_{L_{0}}^{L_{f}}\frac{1}{\dot{s}(s)}\left(1+{\left(\frac{v_{\delta }}{{\Delta w}_{\delta }^{2}{\dot{\delta }}_{max}}\right)}^{2}+{\left(\frac{v_{W}}{{\Delta w}_{W}^{2}{\dot{W}}_{max}}\right)}^{2}\right)ds$$

Each of these three terms was weighted differently with a coefficient (i.e., 1/Δ*w_v_,* 1/Δ*w_n_*, 1/Δ*w_δ_* and 1/Δ*w_W_*, for the longitudinal speed *v*, the lateral displacement *n*, the rate of change in the steering angle $\dot{\delta }$ and the rate of change in the power output $\dot{W}$) in the cost function. Placing a penalty on the rate of variation in the state variables is analogous to penalising the “jerk” in optimal control formulations, a concept frequently employed in motor control modelling [29,30]. In the context of the bike–rider system, this approach helps ensure smooth transitions in steering and power output, reflecting realistic rider behaviour while maintaining numerical stability of the optimisation.

Some constraints were imposed to the optimal control problem. The steering angle *δ* and the power output *W* were constrained between their maximal and minimal values (i.e., *δ*_max_, *δ*_min_, *W_max_* and *W_min_*). The resulting constraints, therefore, were symmetric about zero:

(16) $$-1\leq \delta_{n}(s)\leq 1$$

(17) $$-1\leq W_{n}(s)\leq 1$$

The maximal power output was reduced to zero for roll angles greater than 15*°* (this is because cyclists usually do not pedal while cornering).

Initial values for all the state variables were set to zero, except for the longitudinal velocity, which was set with the initial velocity provided by the GPS (by means of the Physics Toolbox Sensor suite application, ver. 2021.04). The software Maple (Maplesoft, ver. 2018) and MBSymba [31] toolbox were used to formulate and manipulate the equations of the dynamic system. The XOptima toolbox and the software PINS (ver. 1.4.5) [17] were used to formulate and then solve the optimal control problem. The distance between the nodes of the mesh of the optimal control problem was set to 0.5 m.

### 2.4. The Courses

The courses selected for the data collection were characterised by the following:

- Course 1: was characterised by a total length of 5.8 km, a total elevation loss of 263 m, three fast S-turns, one hairpin turn with decreasing radius, two 90-degree turns, one fast long turn, a longer flat segment, and a final roundabout. The course profile and map can be found at the following link: https://www.google.com/maps/d/u/1/edit?mid=1yeyWb-_jFGclNPZM-kwn3Fhiv2wWfq9F&usp=sharing (accessed on 11 November 2025)
- Course 2: was characterised by a total length of 5 km, a total elevation loss of 288 m, three fast S-turns, eight hairpin turns, two 90-degree turns, and one fast long turn. The course profile and map can be found at the following link: https://www.google.com/maps/d/u/1/edit?mid=19sOCsULkUimzP-Xkp9uNyr-lITbSMZfj&usp=sharing (accessed on 11 November 2025)

An amateur cyclist (i.e., average distance cycled in the last 5 years: 2500 km/year) was asked to complete the courses on separate days. Asphalt was dry and of similar temperature and humidity, and non-windy conditions were checked before each course. Because there was no cycling lane, the cyclist was asked to keep to the right lane to avoid dangerous collisions with incoming vehicles. The cyclist knew the courses in advance and was asked to complete the course at a self-selected speed.

### 2.5. Experimental Data Collection

A road bicycle with aerobars was equipped with a smartphone (Xiaomi Mi 10 lite, Xiaomi, Beijing, China), able to collect synchronised data from the embedded accelerometer (Bosch BMA 490 L, 200 Hz, Bosch Sensortec, Reutlingen, Germany), the gyroscope (Bosch BMG 250, 500 Hz, Reutlingen, Germany), and the dual-frequency GPS receiver (L1/L5 frequencies). A custom-written Matlab (Mathworks, ver. 2020a) was used to process the GPS data points and compute the parameters of the trajectory clothoids, by implementing the library of functions by Bertolazzi and Frego (https://it.mathworks.com/matlabcentral/fileexchange/64849-ebertolazzi-clothoids, accessed on 11 November 2025) [32]. The smartphone was fixed to the bicycle frame horizontally on the top tube at the junction with the seat tube. This position was selected because it was near enough to the actual centre of mass of the system. The three axes of the inertial measurement unit (IMU) (i.e., *x*_IMU_, *y*_IMU*,*_ and *z*_IMU_, Figure 1) were pointing forward, leftward and upward. The accelerometer provided three accelerations along the three axes of the IMU: a*_xIMU_, a*_yIMU_ and a*_zIMU_. The accelerometer also acted as an inclinometer to provide the experimental pitch ϑ^*^ (i.e., proxy for the angular displacement of the bike-rider system about its transverse axis), roll φ* (i.e., proxy for the roll angle: the angular displacement of the bike-rider system about its longitudinal axis), and yaw ψ* (i.e., proxy for the yaw angle: the angular displacement of the bike-rider system about its normal axis). The gyroscope provided three angular rates along the three axes of the IMU: ω*_xIMU_, ω*_yIMU_ and ω*_zIMU_. The Physics Toolbox Sensor suite application [33] for Android was used to log and then export the experimental data. The data from the IMU were logged with a non-constant sampling time of 0.0092 ± 0.01 s. It has been noticed that the GPS data were logged with a slower frequency of 1 ± 0.01 s and with an average delay of 0.9 s with respect to the data from the IMU. This delay has been considered, and signals have been correctly aligned. GPS data points were written in a *kml* file, and they were used to estimate the road slope *β* by means of the GPS visualizer online tool (gpsvisualizer.com). Experimental longitudinal speed v* was also computed from GPS data.

One big issue with measuring/estimating the roll angle with an accelerometer is that it can be used to estimate the roll angle only during steady-state cornering, i.e., a condition where a trajectory with constant radius is negotiated at constant speed and constant yaw rate. The method proposed by Boniolo et al. [7] was used here to get closer to the actual conditions:

(18) $$\varphi_{b}^{*}=\varphi_{LF}^{*}+\varphi_{HF}^{*}$$

where φ*_LF_ and φ*_HF_ are the low- and high-frequency (smaller and larger of 0.15 Hz, respectively) components of the experimental roll angle φ*. The φ*_HF_ is computed by simple numerical integration of the high-frequency component of the experimental angular rate ω*_xIMU-HF_. The φ*_LF_ is computed by means of the low-frequency components of the experimental longitudinal speed v*_LF_ and ω*_zIMU-LF_ [7].

Experimental longitudinal and lateral accelerations on the road plane were computed as the projections of the accelerations provided by the IMU on the *xy* plane (Figure 1).

Differences between the values provided by the model and the IMU experimental data were computed for the roll angle and the longitudinal and lateral accelerations. The root mean square error (RMSE) was used to express the magnitude of these differences.

### 2.6. Modelling Assumptions

Like any physically grounded modelling work, this research requires a number of explicit and implicit assumptions spanning the modelling, numerical, and experimental domains. The most fundamental assumption is that the interaction between the rider and the bicycle can be represented by means of an *optimal control system*, where the steering and power output act as control inputs driving the model to reproduce the measured GPS trajectory. In this formulation, the control process implicitly accounts for the rider’s continuous adjustments to maintain balance and trajectory tracking, thus approximating realistic handling behaviour within a physically consistent framework.

Other assumptions related to the modelling choices and to the experimental design are listed here. (i) The system is modelled as an inverted pendulum with a single dominant roll degree of freedom, neglecting complex body–bike coupling motions (e.g., upper body sway, steering compliance, frame flex, wheel deformation). (ii) The gyroscopic and inertial coupling effects are neglected: the stabilising or destabilising effects of wheel gyroscopic precession and steering inertia are omitted for tractability. (iii) The frame, fork, and rider are assumed rigid, ignoring energy dissipation through structural compliance or soft tissues. (iv) The tyre forces are implicitly modelled through lateral and longitudinal acceleration constraints, without explicitly modelling tyre slip, saturation, or camber effects. (v) Rider and bicycle masses, moments of inertia, and geometry are treated as fixed and not speed-dependent or configuration-dependent. (vi) The cost function in the optimal control formulation (Equations (12)–(15)) penalises deviations and rates of change (akin to jerk minimisation), implicitly assuming smooth, human-like control behaviour. (vii) The control problem is regularised to ensure convergence; non-linear or discontinuous dynamics (e.g., tyre slip, non-smooth steering) are excluded for solvability. (viii) The trajectory is discretised with a fixed spatial step (e.g., 0.5 m), assuming this resolution adequately captures dynamic variations without excessive computational cost. (ix) The model assumes that the optimal control process itself acts as a low-pass filter on noisy GPS data, substituting for explicit signal filtering. (x) The model was calibrated and validated on a single amateur cyclist on road descents, assuming representativeness of general rider dynamics. (xi) Weights in the cost function and some numerical tolerances were determined empirically after extensive trial and error, as no theoretical reference exists for this specific problem. (xii) The data sampling frequency and positioning accuracy were considered adequate to capture curvature dynamics (spacing between GPS points can affect curvature and roll angle reconstruction). (xiii) IMU signals are assumed accurate enough for validation despite known biases and drifts; the emphasis is on comparative trends rather than absolute precision.

## 3. Results

**Figure 2:**
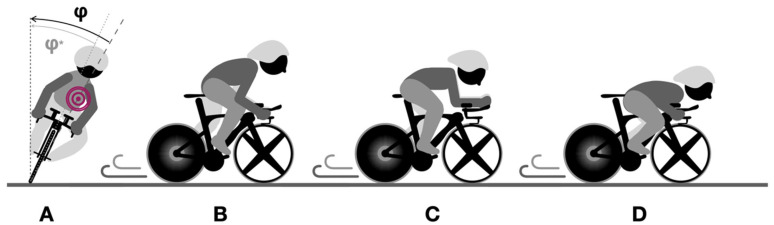
Schematic representation of the most common cycling positions adopted by the cyclist in this study. Red circle represent the centre of mass of the bike-rider system. (**A**) Cornering position where the roll angle of the actual rider–bicycle system *φ,* and the roll angle φ^*^ measured by the inertial measurement unit (IMU) are reported. (**B**) Standing position. (**C**) Aero position. (**D**) Descending position. It is worth highlighting that the mathematical model could not take into consideration these variations in the cyclist’s posture.

Notably, across the different segments of the courses, the cyclist mainly adopted four different positions, which are qualitatively presented in Figure 2.

**Figure 3:**
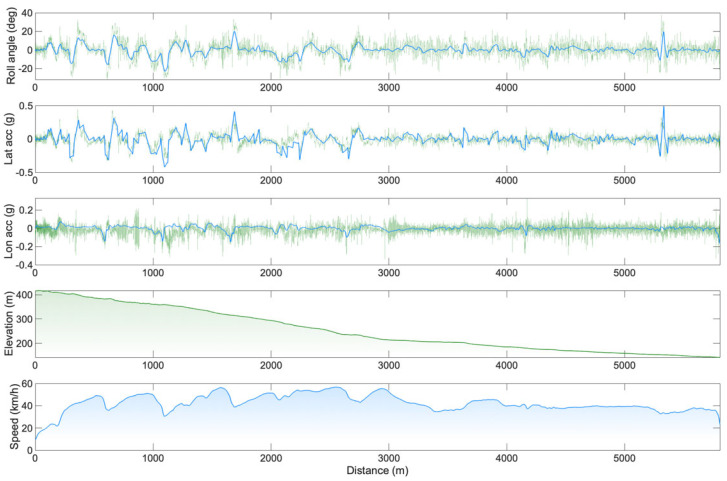
Comparison between simulated (blue) and experimental data (light green) for the first course. From top to bottom: roll angle, lateral acceleration, longitudinal acceleration, elevation, and speed.

Course 1: In total, 151 clothoids were used to fit the GPS experimental data points. The average difference between the fitting clothoids and the experimental points was 0.05 ± 0.049 m, and the maximal error was 0.65 m. The course was completed in 8′39″ with an average speed of 42.2 km/h. For the entire course, an RMSE of 6.5° has been computed for the roll angle estimation, and the RMSE values for the longitudinal and lateral accelerations were 0.06 g and 0.07 g, respectively. Across the 200 m, including the roundabout, the RMSE changed to 9.5°, 0.05 g, and 0.1 g for the roll angle, longitudinal, and lateral accelerations, respectively. Across the 200 m, including the fast 90-degree turn, the RMSE was 8.2°, 0.07 g, and 0.1 g for the roll angle, longitudinal, and lateral accelerations, respectively. An example of the comparisons between simulated and experimental data on the entire course is reported in Figure 3.

**Figure 4:**
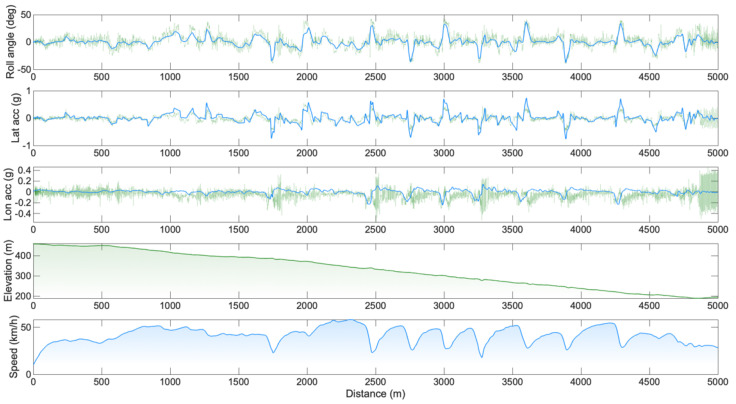
Comparison between simulated (blue) and experimental data (light green) for the second course. From top to bottom: roll angle, lateral acceleration, longitudinal acceleration, elevation and speed. In the very last portion of the signal, a strong pedalling action generating sharp lateral accelerations can be noticed.

Course 2: In total, 130 clothoids were used to fit the GPS experimental data points. The average difference between the fitting clothoids and the experimental points was 0.12 ± 0.13 m, and the maximal error was 1.1 m. The course was completed in 7′36″ with an average speed of 41.5 km/h. For the entire course, an RMSE of 8.7° has been computed for the roll angle estimation, and the RMSE values for the longitudinal and lateral accelerations were 0.1 g and 0.13 g, respectively. Across the 200 m, including one of the hairpin turns, the RMSE changed to 12.3°, 0.12 g, and 0.17 g for the roll angle, longitudinal, and lateral accelerations, respectively. Examples of the comparisons between simulated and experimental data on the entire course are reported in Figure 4.

## 4. Discussion

The methodology presented in this paper has been conceived for road cycling races and for fast descending sections. Data collected on two different courses, which could constitute typical segments in road cycling races, were used to assess the accuracy of the estimations in roll angle, longitudinal, and lateral accelerations. Provided that only GPS data were used in this research, an RMSE range of 6.5–12.3° in the estimation of the roll angle constitutes an encouraging result, but improvements are possible. In comparison with the errors reported by Baniolo et al. [7] (5° in the roll angle estimation), the error is almost double but still deemed acceptable for outdoor applications. Boniolo et al. [7], in their experimental work, declared an additional error of 2° due to the tyre cross-section of their vehicle (i.e., a motorcycle). It can be assumed that, in bicycles, the tyre cross-section introduces a negligible error due to the tyre cross-sectional area (for a bicycle, the contact point can be assumed to lie on the bicycle symmetry plane [8]).

In the present work, the IMU was fixed to the frame of the bike. Another option would have been to fix the IMU to the body of the cyclist. Placing the IMU on the frame of the bike can provide abrupt lateral accelerations because of the cyclist’s pedalling action. During actual cycling races, cycling computers are placed on the handlebars. This is because riders can benefit from visual feedback by checking the display of the computer. Even if cycling computers were equipped with an IMU, estimating the roll angle with an IMU fixed on the handlebars would be challenging. This is because there would be an additionally complex influence of the steering angle. Considering these limitations, the ranges of the RMSE reported for the longitudinal (0.05–0.12 g) and the lateral (0.1–0.17 g) accelerations can be considered an encouraging result. In a recent paper, Zignoli et al. [6] showed that the magnitude of accelerations is associated with performance in downhill sections in road cycling. Inter-individual differences between cyclists might be compatible with the error reported here. Evaluating whether this methodology can be used to assess the difference between expert and novice riders or to classify riders by bike-handling ability was deemed out of scope. This might become the subject of the next research work.

Graphs reported in Figure 3 and Figure 4 can be used to spot the sections of the course where the error in the estimation was maximum. Most of the errors in the estimation could be due to the changes in the riding position. In these regards, fixing the IMU on the body of the cyclist would include different information due to the cyclist’s body displacement [12]. The most problematic positions were the first and second riding positions (Figure 2A and 2B). The first was the cornering position: the cyclist needed to move the hands from the aerobars to the hoods to improve bicycle manoeuvrability. In this position, it is likely that the upper body motion could move the centre of mass of the system away from the plane of symmetry of the vehicle, where the IMU was positioned (i.e., discrepancy between measured roll angle φ^*^ and actual roll angle of the rider–bicycle system φ). Another problematic position is reported in Figure 2B. This standing position was used by the cyclist to deliver high torque values and accelerate after the corners. In this position, the IMU likely sensed strong accelerations due to the pedalling action (these strong accelerations can be easily spotted in Figure 4, towards the end of the course).

The greater RMSE values reported for the small segments (i.e., hairpin turns or roundabouts, clearly discernible in Figure 3 and Figure 4) reinforce the idea that the estimation of the roll angle and the road-plane accelerations might be problematic in slow segments, where cyclists are asked to brake, turn frequently, and re-accelerate. Two other common riding positions are reported in Figure 2C and 2D. In these positions, the centre of mass of the system was better aligned with the plane of symmetry of the vehicle. In the aero C position, the cyclist was pedalling with a steady action, and the arms were placed on the handlebars. In the descending D position, the cyclist was not pedalling, the chest was placed on the handlebars, and the hands were placed on the hoods. This methodology is expected to be particularly accurate and reliable on fast road segments (where riding positions C and D are commonly adopted), where additional complexities not considered in the rider–bicycle dynamic model could only play a marginal role.

Another potential limitation of this methodology is that the road geometry has been considered flat, without any banking. The information about this angle is particularly complex to retrieve, especially in long road segments. Unfortunately, no information is available on the amount of banking in the two courses. This adds more uncertainty to the estimation of the roll angle, which is affected by the road banking angle. The model developed by Fitton and Symons for track cycling [34], for instance, takes the banking angle into consideration, but it has been developed mainly for track cycling, where racing sections are considerably shorter than in road cycling.

As with any computational model, the development of the bicycle–rider system involves a fundamental trade-off between accuracy and solvability. Over several years of iterative refinement and trial-and-error testing, we identified a set of assumptions that allowed the optimal control algorithm to converge to physically meaningful solutions while capturing the essential dynamics of the system. Some of these assumptions are specific to this study and, due to their nuanced nature, are not directly found in the literature. While the current model strikes a balance between realism and numerical tractability, future work could explore the inclusion of additional non-linear terms or secondary dynamic effects to further enhance model fidelity without compromising convergence.

Another intrinsic limitation stems from the quality of the input data. GPS and IMU signals are inherently noisy, which can affect the accuracy of reconstructed states. However, the optimal control formulation employed in this study inherently mitigates high-frequency noise: the model acts as a physics-based low-pass filter, fitting the measured data within the constraints of the system dynamics. This filtering effect explains why the current method is expected to perform particularly well on downhill routes, where GPS points are more regularly spaced and dynamic variations are smoother. Future work should investigate how the method performs under more variable terrain, lower speeds, and higher-frequency motion to ensure robustness across diverse cycling conditions.

Finally, while the present study establishes a methodological framework for GPS-based estimation of roll angle and accelerations, it does not include direct benchmarking against alternative estimation methods. Comparisons with other algorithms, including vision- or gyroscope-based approaches, as well as formal statistical validation across larger and more diverse datasets, remain important avenues for future development. Extending the framework to include performance comparisons and larger populations will help quantify its reliability, generalisability, and potential advantages over existing approaches.

The accuracy of this methodology might not be sufficient to provide a reliable source of information for the design of safety action controllers. Safety controllers usually operate in high-stakes environments, and they need accurate and reliable information about the state of the bike-rider system. However, the accuracy of the present methodology might be sufficient to develop indices for the different standard manoeuvres and classify good and bad handling in cyclists, e.g., by examination of the adherence ellipse, as it was recently performed in [6]. It is worth highlighting that this methodology only relies on GPS data and, therefore, can be easily accessed by a large pool of practitioners. In the world of sports sciences, quantitative handling research on road cycling races is very limited. The use of this methodology in the development of a set of guidelines for different levels of bike-handling ability could help manufacturers design racing bicycles that are conceived for specific groups, such as ‘rookie’, ‘transitional’ or ‘daredevil’. Coaches and athletes could use this tool to run data analyses in post-processing and test/develop specific training for technical skills development.

## 5. Conclusions

With the goal of developing a standard, cost-effective tool for assessing bike handling skills in road cycling training and races, a rider–bicycle model was deployed within an optimal control framework to estimate roll angle as well as longitudinal and lateral accelerations using GPS data only. The accuracy of the roll angle estimations slightly exceeds that reported for other similar methods that exploit alternative cost-effective hardware solutions (e.g., onboard gyroscopes or accelerometers). Importantly, the methodology presented in this paper relies solely on GPS data, which can facilitate the broader deployment of this approach on cycling computers. This would enable a rapid dissemination of more in-depth bike-handling performance metrics in road cycling.

## Data Availability

Data associated with this manuscript will be made fully available upon reasonable request to the author.

## Abbreviations

Numerical Values and List of Symbols	
*a*_xmax_ = *a*_ymax_ = 9.81 m/s^2^	longitudinal and lateral maximal accelerations
*g* = 9.81 m/s^2^	constant of gravity
*h* = 1 m	height of the centre of mass from the road surface
*k*_v_ = ½ *A*_f_ *C*_D_ *ρ* = 0.15 kg/m	air drag force coefficient
*m* = 85 kg	cyclist’s body mass + bicycle mass
*n*_0_ = 0 m	initial lateral displacement
s_0_ = *L*_0_ = 0 m	initial longitudinal position
${\dot{W}}_{max}$= 50 W/s	maximal rate of power output
*w*_D_ = *π* rad	wind direction
*A*_f_ = 0.35 m^2^	frontal area
*C*_D_ = 0.7	drag coefficient
*C*_rr_ = 0.004	rolling friction coefficient
*I*_X_ = 77 kgm^2^	inertia of the system along the longitudinal direction
*L* = 1.4 m	wheelbase
*L*_f_ = XX m	course length (depending on the course)
*V_w_* = 0 m/s	wind speed
*W*_n0_ = 0	initial normalised power output
*W*_max_ = 1200 W	maximal power output
*a*_0_ = 0 rad	initial heading
Δw_n_ = 1	weight of the lateral displacement in the objective function
Δw_v_ = 1	weight of the error with the reference velocity in the objective function
Δw_w_ = 10	weight of the rate of change in the power output in the objective function
Δw*_δ_* = 10	weight of the rate of change in the steering angle in the objective function
*δ*_max_ = 0.52 rad	maximal steering angle
${\dot{\delta }}_{max}$= 0.52 rad/s	maximal steering velocity
*δ*_n0_ = 0	initial normalised steering angle
*φ*_0_ = 0 rad	initial rolling angle
${\dot{\varphi }}_{0}$= 0 rad/s	initial roll rotational velocity
*ρ* = 1.23 kg/m^3^	air density
